# Electric Fan Use With Dehydration in Extreme Heat and Humidity: A Randomized Crossover Trial

**DOI:** 10.1001/jamanetworkopen.2025.26701

**Published:** 2025-08-13

**Authors:** Connor Graham, Lily Hospers, Ollie Jay

**Affiliations:** 1 Heat and Health Research Centre, Faculty of Medicine and Health, University of Sydney, Camperdown, New South Wales, Australia

## Abstract

This randomized crossover trial evaluates whether hydration levels modify the effects of fan use on cardiovascular strain, body temperature, and thermal comfort after 3 hours of heat exposure.

## Introduction

Heat extremes have devastating impacts on human health. More than 60 000 heat-related deaths occurred in the 2022 European summer,^1^ while record-breaking temperatures across North America in 2021 caused more than 750 heat-related deaths.^2^ Most extreme heat decedents do not have air conditioning but often own electric fans.^2^ Fan use can reduce heat-related elevations in thermal and cardiovascular strain at temperatures up to approximately 39 to 40 °C.^3,4^ In hotter conditions, fans should be turned off, as they can worsen heat stress because accelerated convective heat gain outweighs increased sweat evaporation.^5^ Progressive dehydration during heatwaves is common. Associated sweating impairments could subsequently alter the cooling effect of fan use. This study examined whether dehydration modifies the effect of electric fans on cardiovascular and thermal strain as well as thermal comfort during a 3-hour hot-humid heat exposure.

## Methods

Following University of Sydney ethical approval, 20 participants (eTable in Supplement 1) were recruited, gave written informed consent, and enrolled (Figure). Reporting follows CONSORT guidelines. For 5 days before their first trial, participants established a hydration baseline, measuring daily nude body mass after waking and voiding their bladder and urine specific gravity from a first-pass midstream sample. In a randomized crossover design, participants were allocated to complete four 3-hour experimental trials, each on separate days (Supplement 2). Wearing shorts and a cotton vest, participants sat in a climatic chamber regulated at 39.2 ± 0.7 °C with 49% ± 4 relative humidity. These conditions represent the environmental threshold that fans can become detrimental for optimally hydrated healthy adults.^5^ In 2 euhydrated trials, participants consumed fluids according to daily recommendations for 24 hours prior and drank a prescribed volume of approximately 37 °C water throughout their heat exposure, maintaining euhydration. In 2 dehydrated trials, participants did not consume fluids or food containing more than 75% water for 24 hours prior and drank no fluid throughout their heat exposure. Each hydration condition was tested with and without a 44.5-cm fan, placed 1.5 m away and facing the participant (air velocity, 2.5 m/s). Primary outcomes were rectal temperature (Covidien) in degrees Celsius, heart rate (HR) (Polar) in beats per minute, and whole-body sweat rate in grams per hour determined using the 180-minute pretrial to posttrial change in body mass (Mettler), corrected for fluid consumption. Secondary outcomes were thermal discomfort and sensation rated on a 100-mm visual analogue scale (ASHRAE) and thirst gauged by summing three 100-mm visual analogue scales. The effect of hydration (euhydrated/dehydrated) on the influence of fan use (on/off) for all outcomes was assessed using a linear mixed model analysis with Sidak post hoc testing (SPSS version 29.0.1 [IBM Corp]) (Supplement 2).

**Figure.**
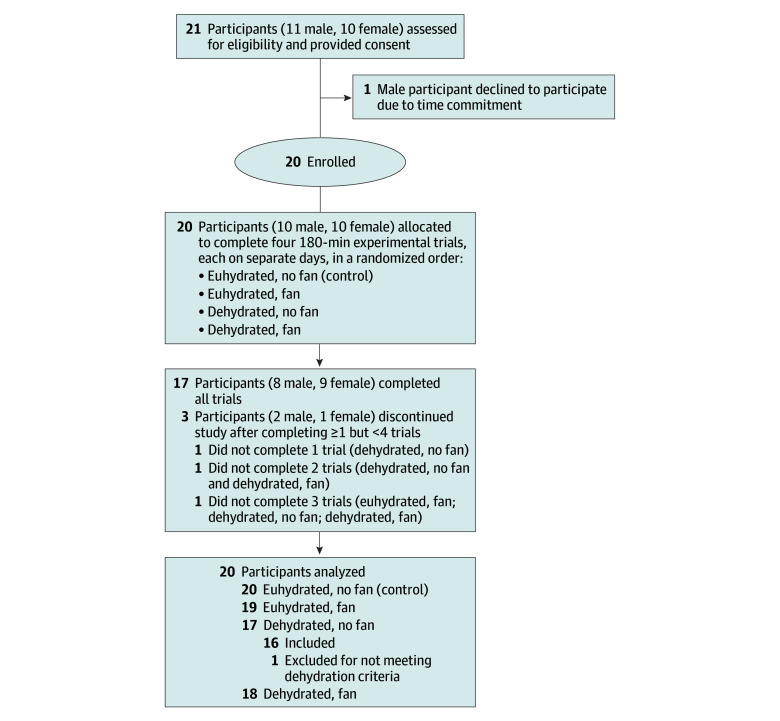
Flow of Participants Through the Study Sample size was determined via power calculation using an α of .05, a β of 0.1, and an effect size of 0.78.

## Results

All baseline and end-trial outcome variables and between-group comparisons of end-trial values are displayed in the Table. No adverse events were reported in the study. There was an interaction between fan use and hydration status for end-trial HR (*P* = .02) as well as thermal comfort (*P* = .006) and sensation (*P* = .007). The effect of fan use on end-trial core temperature, whole-body sweat rate, and thirst was similar between dehydration and euhydration. Irrespective of dehydration, whole-body sweat rate was greater with fan use (eg, dehydrated, fan vs no fan: mean difference, 125 [95% CI, 108-142] g × h^−1^; *P* = .005). Irrespective of fan use, dehydration drastically worsened all markers of thermal, cardiovascular, and subjective strain (eg, HR for no fan, dehydrated vs euhydrated: mean difference, 17 [95% CI, 13-20] beats/min; *P* < .001).

**Table.** Outcomes at Baseline and at the End of Heat Exposure, With Between-Group Comparisons of Change in Outcome Variables

Condition	First-pass USG upon arrival, AU	Body mass change, %	Rectal temperature, °C	Heart rate, beats × min^−1^	Whole-body sweat rate, g × h^−1^	Thermal sensation, mm^a^	Thermal discomfort, mm^b^	Thirst, mm^c^
**Last 5-min at baseline before heat exposure, mean (SD)**								
Euhydrated, no fan	1.019 (0.008)	0.04 (0.76)^d^	37.0 (0.3)	74 (10)	NA	44 (8)	5 (12)	46 (47)
Euhydrated, with fan	1.017 (0.009)	0.46 (0.99)^d^	37.0 (0.4)	74 (11)	NA	42 (12)	3 (8)	28 (35)
Dehydrated, no fan	1.031 (0.005)	−1.15 (0.61)^d^	37.1 (0.3)	76 (13)	NA	44 (8)	7 (15)	187 (68)
Dehydrated, with fan	1.032 (0.008)	−1.23 (0.68)^d^	37.1 (0.2)	78 (13)	NA	43 (12)	8 (12)	178 (81)
**Last 5-min of heat exposure, mean (SD)**								
Euhydrated, no fan	NA	−0.19 (0.81)^e^	37.3 (0.2)	81 (9)	220 (28)	83 (12)	53 (21)	33 (38)
Euhydrated, with fan	NA	0.13 (0.84)^e^	37.3 (0.2)	81 (11)	354 (64)	75 (10)	36 (17)	27 (51)
Dehydrated, no fan	NA	−2.25 (0.61)^e^	37.7 (0.2)	92 (12)	198 (29)	90 (8)	73 (19)	267 (47)
Dehydrated, with fan	NA	−2.72 (0.76)^e^	37.7 (0.2)	97 (12)	323 (41)	89 (9)	77 (22)	281 (36)
**Group differences in the change from baseline to end trial, mean difference (95% CI)**								
Euhydrated, fan vs no fan	NA	NA	0.0 (0.0 to 0.1)	0 (−3 to 4)	135 (119 to 151)	−9 (−13 to −4)	−17 (−27 to −6)	−6 (−30 to 19)
*P* value	NA	NA	.36	.89	<.001	<.001	<.001	.64
Dehydrated, fan vs no fan	NA	NA	0.0 (0.0 to 0.1)	5 (1 to 9)	125 (108 to 142)	−1 (−6 to 4)	4 (−7 to 15)	20 (−17 to 56)
*P* value	NA	NA	.28	.02	.005	.55	.53	.28
No fan, dehydrated vs euhydrated	NA	NA	0.3 (0.2 to 0.4)	12 (8 to 15)	−21(−37 to −4)	7 (2 to 12)	20 (5 to 35)	235 (209 to 261)
*P* value	NA	NA	<.001	<.001	.02	.004	.008	<.001
Fan, dehydrated vs euhydrated	NA	NA	0.3 (0.2 to 0.4)	17 (13 to 20)	−28 (−45 to −12)	15 (10 to 20)	40 (30 to 51)	255 (229 to 281)
* P* value	NA	NA	<.001	<.001	.001	<.001	<.001	<.001

Abbreviations: AU, arbitrary unit; NA, not applicable; USG, urine specific gravity.

^a^ Scored on 100-mm visual analogue scale where 0 indicates cold, 50 indicates neutral, and 100 indicates hot.

^b^ Scored on 100-mm visual analogue scale where 0 indicates not uncomfortable and 100 indicates very uncomfortable.

^c^ The sum of 3 thirst indicators were each separately scored on 100-mm visual analogue scales according to the following questions: (1) How thirsty do you feel right now? (where 0 indicates not thirsty at all and 100 indicates very thirsty); (2) How dry does your mouth feel right now? (where 0 indicates not at all dry and 100 indicates very dry); (3) If you would like a drink, how pleasant would it be to drink water right now? (where 0 indicates I do not want a drink and 100 indicates very pleasant.)

^d^ Body mass change measured from 24-hour before trial to baseline.

^e^ Body mass change measured from baseline to end of trial.

## Discussion

In this study, fan use worsened cardiovascular strain during a 3-hour hot-humid heat exposure with dehydration, but no effect of fan use was observed with euhydration, unlike in our previous study.^3^ Benefits of fans for improving thermal sensation and relieving discomfort with euhydration were eliminated by dehydration. Regardless of fan use, heat-related increases in HR and core temperature were 2.0- to 2.5-fold greater when dehydrated, underscoring the importance of hydration during heatwaves. Fans accelerate sweat losses by approximately 60%, and their use when dehydrated may worsen cardiovascular strain and not improve comfort. Limitations include assessing young healthy participants in one condition. Future research should assess fans combined with self-dousing^6^ and test older participants with varying health and medication status across different conditions.

## Notes

Supplement 1. **eTable.** Participant Characteristics

Supplement 2.
Trial Protocol

Supplement 3.
Data Sharing Statement
