# Supplementary material for: Electric Fan Use With Dehydration in Extreme Heat and Humidity: A Randomized Crossover Trial
Source: JAMA Netw Open. 2025 Aug 13;8(8):e2526701. doi: 10.1001/jamanetworkopen.2025.26701 (PMC12351413; doi:10.1001/jamanetworkopen.2025.26701)
Supplement: Supplement 1. — eTable. Participant Characteristics [file jamanetwopen-e2526701-s001.pdf]

Supplemental Online Content

Graham C, Hospers L, Jay O. Electric fan use and hydration in extreme heat and humidity: a randomized crossover trial. *JAMA Netw Open.* 2025;8(8):e2526701. doi:10.1001/jamanetworkopen.2025.26701

eTable. Participant Characteristics

This supplemental material has been provided by the authors to give readers additional information about their work.

© 2025 Graham C et al. *JAMA Network Open*.

eTable. Participant Characteristics

**Participant characteristics, Mean (SD) [Range]**	
Participants, n	20 (10♀/10♂)
Age, y	28 (5) [20-34]
Nude body mass, kg †	71.5 (11.1) [41.8-87.7]
Height, m	1.72 (0.09) [1.56-1.87]
Body surface area, m^2	1.84 (0.19) [1.37-2.10]
**5-day hydration baseline, Mean (SD)**	
Mean daily mass fluctuation (%)	0.40 (0.16)
Average first pass USG (AU)	1.022 (0.006)

© 2025 Graham C et al. *JAMA Network Open*.
