# Supplementary material for: Electric Fan Use With Dehydration in Extreme Heat and Humidity: A Randomized Crossover Trial
Source: JAMA Netw Open. 2025 Aug 13;8(8):e2526701. doi: 10.1001/jamanetworkopen.2025.26701 (PMC12351413; doi:10.1001/jamanetworkopen.2025.26701)
Supplement: Supplement 2. — Trial Protocol [file jamanetwopen-e2526701-s002.pdf]

Final protocol

Dehydration Alters the Effect of Electric Fans on Cardiovascular Strain in Extreme Heat and Humidity

Funding organizations

National Health and Medical Research Council (NHMRC) Investigator Grant (Holder: O.Jay)

Principal investigator

Ollie Jay (PhD)

Collaborators

Connor Graham (PhD)

Lily Hospers (PhD)

Research site:

Thermal Ergonomics Laboratory

Heat and Health Research Centre

Susan Wakil Health Building

University of Sydney

Camperdown, Sydney, NSW

Australia

1 - Definition of the research problem and relevant literature

The exponential growth in extreme heat events subsequent to global climate change is leading to increasing heat related morbidity and mortality around the world [1-5]. The impact, whilst global, is unevenly distributed, with certain demographics suffering much more than others during heatwaves. Those with low socio-economic status are one such demographic [6].

Paradoxically, the presence of, and capacity to afford, air conditioning inside the home decreases risk of ill-health during heatwaves significantly [6] whilst increasing energy usage markedly, thereby further contributing to global climate change [7]. Conversely, those without an air-conditioner, or who are unable to afford to turn it on suffer worse during heatwaves, and must rely on low-cost, and often inadvertently low-emission, cooling interventions. One such intervention that consumes ~45 times less power than air conditioning is the electric fan [8]. However, national [9, 10] and global groups, including the World Health Organisation [11] still advise avoiding electric fan use in environments where ambient temperature exceeds skin temperature (~35˚C), stating that in these settings they exacerbate strain, in spite of recent evidence dispelling this common belief [12].

Contrasting the stigma surrounding fan use in the heat is the recent work of Morris et al. [12] which demonstrated that fan use can mitigate cardiovascular strain, whilst not worsening thermal strain in ambient temperatures of 40˚C. Morris et al. [12] demonstrated the relationship between humidity and the effectiveness of fan use, as strain was worsened by fan use in very dry conditions but mitigated in more humid environments. This research has laid the foundation for global change in the advice given to the public about electric fan use during heatwaves by dispelling the myth that there is a critical temperature beyond which fans stop working. However, there are still concerns about the use of fans when coupled with dehydration which must be addressed in order to confirm utility of electric fans in real life heatwave contexts, during which mild dehydration may be present. In the work of Morris et al. participants were euhydrated throughout [12].

Fundamentally, fans off-set thermal strain by increasing the rate at which sweat evaporates from the skin surface, which is our best heat dissipation mechanism [13]. In dry environments, sweat already evaporates readily; this is why fans are not helpful in dry heatwave simulations, but in humid contexts, sweat does not evaporate as readily, therefore the assistance given by

the forced airflow of an electric fan mitigates thermal strain [12]. Dehydration can cause reductions in sweat output, which may reduce or even reverse the advantage of fan use [14]. If air warmer than the skin surface is forced over dry skin (subsequent to dehydration related decrements in sweat output) it may lead to convective heat gain, which would in fact worsen thermal strain.

The degree of sweat output reduction is directly related to the level of dehydration; it is as yet unknown whether the amount of dehydration which may occur in a heatwave (~2-3% total body weight) causes a significant enough reduction in sweat rate to impact the utility of electric fan use and if so, how great this impact is. Ultimately, it is important to understand this relation as it may impact public health guidance surrounding electric fan use in heatwaves. It is therefore the aim of this study to quantify the effect of a realistic level of dehydration on electric fan utility during an extreme heat event with a view towards informing publicly available heatwave advice.

2 - Objectives and evaluation criteria

2.1. Primary objective

To examine whether dehydration modifies the effect of electric fan use on thermal and cardiovascular strain, and thermal comfort during a 3-h exposure to a simulated peak hot-humid heatwave condition.

2.4 *Outcome variable summary*

**Outcome variable**	**Analysis timepoint(s)**
Rectal temperature (°C)	End of heat exposure (average of last 5-min of 3-h heat exposure)
Heart rate (beats·min^{-1})	End of heat exposure (average of last 5-min of 3-h heat exposure)
Whole-body sweat rate (g·h^{-1})	Cumulative total sweat loss during heat exposure divided by total heat exposure time
Thermal sensation (mm)	Final rating at end of heat exposure
Thermal discomfort (mm)	Final rating at end of heat exposure
Thirst (mm)	Final rating at end of heat exposure

3 - Methods

3.1 Study design

A repeated measures (crossover) design study requiring all participants to complete one 5-day 'hydration baseline' period, followed by four 3-h passive heatwave exposures (counterbalanced using balanced Latin square design), two whilst well hydrated, two hypohydrated, both with and without an electric fan. Experimental trials will be separated by at least 96 h, and all preceded by a 24-h period of carefully prescribed fluid consumption that will ensure either euhydration or contribute to the development of moderate hypohydration. A crossover design was selected because the lower within-person variation in the human heat stress response compared to between individuals enables better statistical power for the detection of between-condition effects.

Hydration baseline:
To contextualize changes in hydration status throughout the study each participant will undergo a 5-day hydration baseline immediately preceding their first experimental trial. A minimum of 3 d monitoring has previously been demonstrated to provide a valid indication of typical daily body mass fluctuations [15]. The hydration baseline involves collecting a first pass, mid-stream urine sample which will later be used for the purposes of urine specific gravity (USG) analysis with a refractometer (Atago, Japan), as well as a nude body mass measurement with an empty bladder, immediately after waking. Participants will be provided with a portable platform scale (KW4050, @weigh, Australia) and carry out this data collection at the same time of day each morning after waking for 5 days. All urine samples will be stored at 4°C for no more that 48 h before analysis [16].

Main experimental trials:
Following a 5-day hydration baseline, participants will be asked to complete four 3-h passive heatwave exposures (39°C, 50% relative humidity) with all trials separated by a minimum of 4 days, to eliminate any carryover effects, and completed within a 3-week span to ensure no discernible difference in heat acclimatization status. All experimental trials are scheduled to start at 0800 h, with participants twice asked to adhere to a 'high fluid intake' and twice to a 'low fluid intake' protocol in the 24 h leading up to the experimental visit. The aim of the high fluid intake protocol is to ensure the participant arrives well hydrated for their experimental

visit. Based on the work of Armstrong and Johnson (2018) detailing daily fluid requirements of men and women [17], female participants were asked to consume 1.8-2.1 L, and male participants 2.4-2.8 L of water throughout the day before their two euhydrated trails, as well as ~500 ml of water alongside a light meal in the 2 h leading up to the trial. The low fluid intake protocol requires participants to consume no water or any other drink during the 24 h leading up to their two hypohydrated trials, as well as the avoidance of food products with >75% water content, a protocol that has previously resulted in a 24-h body mass reduction of ~2% [18].

In advance of all experimental trials, participants will also be asked to abstain from caffeine and alcohol and to avoid strenuous physical exertion for 24 h. Additionally, participants will be asked to maintain a food diary throughout their first high and low fluid intake protocols and replicate them the second time they undergo each protocol.

The four experimental trials differ firstly in hydration status upon arrival and throughout (euhydrated or hypohydrated) and secondly via the employment of an electric fan, placed 1.5 m from the participant provided constant forced airflow of 2.5 m/s (measured 1.5 m away from the fan) throughout the exposure, equivalent to the high setting on a commercially available pedestal fan. The four experiment trials are therefore:

(1) Euhydration, No-Fan (EUH-NF)
(2) Euhydration, Fan (EUH-FAN)
(3) Hypohydration, No-Fan (HYP-NF)
(4) Hypohydration, Fan (HYP-FAN).

To maintain hydration status throughout the euhydration trials, participants will receive either 2.5 ml/kg (EUH-NF) or 3.5 ml/kg (EUH-FAN) of 37°C water at the 30, 60, 90, 120 and 150-min marks. A greater amount of water is given in the EUH-FAN trial to offset the greater sweating output associated with fan use, compared to no fan, that has been previously observed [12, 19]. No water will be provided or consumed during hypohydration trials.

3.2 Description of the population

To complete this study, young healthy participants (non-smokers with no history of respiratory, metabolic, cardiovascular, blood pressure disease, or of diabetes and not currently on any medication) between the ages of 18 and 40 will be recruited.

3.2.1 Inclusion/Exclusion criteria

Age: All participants must be between 18 and 40 years old.
Medication: Participants must not be currently taking any medication.
Disease History: Participants must not currently have or have had in the last 24 months a respiratory (e.g. COPD, severe asthma), kidney (e.g. kidney disease, acute kidney injury) or metabolic disease (e.g. diabetes, endocrine disorders). Participants must have no history of cardiovascular disease.
Smoking status: Participants must have been non-smokers for >24 months prior to participation in this study.
Fear of needles (trypanophobia): Participants with trypanophobia will be excluded as insertion of the rectal temperature probe may elicit a similar response.

3.2.3 Strategy for recruiting and retaining participants in the study

To recruit participants, a recruitment flyer has been created. This flyer will be printed out and posted around/ circulated at the University of Sydney. Further, we intend to circulate information relating to the study on pre-existing Thermal Ergonomics Laboratory social media accounts including Twitter, Facebook, and Instagram. These social media accounts are already active. Specifically, we intend to circulate an infographic which has been edited for better suitability to phones/ tablets/ computers but contains the same information as the printable flier.

The researchers' contact information will be on these flyers and those individuals who may be interested in participating can contact the researchers at their own discretion.

Initial contact will be made when the participants phone or email the researchers. As such, the opportunity for coercion is minimized. Upon receiving initial contact, a standardized email will be sent to the potential participant: i) outlining inclusion/exclusion criteria; ii) providing an outline of the study details with an attached Participant Information Sheet; iii)

inviting the participant to schedule preliminary visit to learn more about the study and provide informed consent.

3.3 Measurements

Rectal probe: In order to monitor the amount of heat stored in the body the participant will be asked to insert a thin, flexible temperature probe ~12 cm into their rectum. Complete written instruction will be given to the participants on the placement of the rectal probe to ensure their safety and comfort. In brief, they will be asked to place a marker ~12 cm from the tip of the probe using sterile surgical tape. They will then insert the probe until the tape reaches their anal surface. On occasion, the insertion of the probe may cause some mild discomfort and minor irritation; however, this sensation passes within 5 minutes. The participant will be responsible for the insertion of this probe.

Heart rate: Measures of heart rate will be recorded continuously using a two-channel chest band (Polar, Kempele, Finland).

Whole-body sweat loss (WBSL): The participant will be weighed on a platform scale nude in triplicate at baseline, and immediately after the exposure to determine whole body sweat loss.

Resultant dehydration (%DEH): Will be calculated using pre and post body masses.

Whole-body thermal sensation (WBTS): During the trial participants will be asked, every 20 minutes, to rate their whole-body thermal sensation on a 100 mm visual analogue scale that ranks from very cold (like touching an ice cube) to very hot (hottest they have ever felt).

Thermal discomfort: During the trial participants will be asked, every 20 minutes, to rate their thermal comfort on a four-point visual analogue scale which ranges from not uncomfortable to very uncomfortable.

Rating of Thirst: During the trial participants will be asked to rate their thirst, every 20 minutes, on a visual analogue scale. This scale consists of 3 questions:

(i) How thirsty do you feel right now?
(ii) How dry does your mouth feel right now?
(iii) How pleasant would it be to drink some water right now?

For each of these questions' participants mark anywhere on a 100 mm line giving a score out of 100 for each question.

4 - Statistics

4.1 Sample size

To complete this study, we plan to recruit at least 16 young healthy participants (non-smokers with no history or current respiratory, metabolic, and cardiovascular illness/disease, high blood pressure, or diabetes, and not currently on any medication. Male/female participants between the ages of 18 and 40 will be recruited. The number of participants was selected by performing a power calculation using an α of 0.05, a β of 0.1, an effect size of 0.78 calculated from the differences in change in rectal temperature between euhydrated and mildly (1.9% total body weight) dehydrated individuals across a three hour heatwave simulation [14].

4.2 Statistical analysis

The effect of hydration (euhydrated/dehydrated) on the influence of fan use (on/off) for all outcomes will be assessed using a Linear Mixed Model analysis with Sidak post-hoc testing.

Specifically, we will use a restricted maximum likelihood (REML)-based linear mixed model, accounting for repeated measures, including a term for hydration status (euhydrated, dehydrated), a term for condition (no fan use, fan use), and interaction term between hydration status and condition. This model will be used to compare end-trial values in the primary outcome variables: rectal temperature, heart rate, and whole-body sweat rate, and the secondary outcome variables: thermal sensation, thermal discomfort and thirst. Any missing data will be handled under the Missing At Random (MAR) assumption without listwise deletion. This allows the retention of participants who have missing data in one or more conditions. All available data will be used in the estimation of fixed effects.

The significance threshold is set at P=0.05. SPSS Statistics (v29.0.1) will be used for all statistical analysis.

5 - References

[1] Seneviratne, S.I., et al., *Allowable CO2 emissions based on regional and impact-related climate targets.* Nature, 2016. **529**: p. 477.

[2] Kaiser, R., et al., *The effect of the 1995 heat wave in Chicago on all-cause and cause-specific mortality.* Am J Public Health, 2007. **97 Suppl 1**: p. S158-62.

[3] Semenza, J.C., et al., *Excess hospital admissions during the July 1995 heat wave in Chicago.* American Journal of Preventive Medicine, 1999. **16**(4): p. 269-277.

[4] Semenza, J.C., et al., *Heat-Related Deaths during the July 1995 Heat Wave in Chicago.* New England Journal of Medicine, 1996. **335**(2): p. 84-90.

[5] Vandentorren, S., et al., *Mortality in 13 French Cities During the August 2003 Heat Wave.* American Journal of Public Health, 2004. **94**(9): p. 1518-1520.

[6] Zhang, Y., et al., *Risk factors of direct heat-related hospital admissions during the 2009 heatwave in Adelaide, Australia: a matched case–control study.* BMJ open, 2016. **6**(6): p. e010666.

[7] Davis, L.W. and P.J. Gertler, *Contribution of air conditioning adoption to future energy use under global warming.* Proceedings of the National Academy of Sciences, 2015. **112**(19): p. 5962-5967.

[8] Labs, N. Estimating Appliance and Home Electronic Energy Use. 2018 [cited 2018 18 November].

[9] Government, V.S., Heat Health Plan for Victoria, H.a.H. Services, Editor. 2015. p. 36.

[10] Heatwave plan for England - protecting health and reducing harm from severe heat and heatwaves. 2018, Public Health England. p. 43.

[11] Organisation, W.H., Heat-waves: risks and responses. 2004, World Health Organisation. p. 124

[12] Morris, N.B., et al., *The Effects of Electric Fan Use Under Differing Resting Heat Index Conditions: A Clinical Trial*. Annals of Internal Medicine, 2019.

[13] Parson, K., Human thermal environments. Human Thermal Environments, 2003.

[14] Graham, C., Morris, N. B., Harwood, A. E., & Jay, O. (2020). *Ad libitum water consumption off-sets the thermal and cardiovascular strain exacerbated by dehydration during a 3-h simulated heatwave*. European journal of applied physiology, 120, 391-399.

[15] Cheuvront, S.N., et al., *Daily body mass variability and stability in active men undergoing exercise-heat stress*. International journal of sport nutrition and exercise metabolism, 2004. 14(5): p. 532-540.

[16] Adams, J., et al., *The effect of storing temperature and duration on urinary hydration markers.* International journal of sport nutrition and exercise metabolism, 2017. 27(1): p. 18-24.

[17] Armstrong, L.E. and E.C. Johnson, *Water intake, water balance, and the elusive daily water requirement.* Nutrients, 2018. 10(12): p. 1928.

[18] Phillips, P.A., et al., *Reduced thirst after water deprivation in healthy elderly men.* New England Journal of Medicine, 1984. 311(12): p. 753-759.

[19] Ravanelli, N.M., et al., *Heart rate and body temperature responses to extreme heat and humidity with and without electric fans*. JAMA, 2015. 313(7): p. 724-725.
