# Supplementary material for: Electric Fan Use With Dehydration in Extreme Heat and Humidity: A Randomized Crossover Trial
Source: JAMA Netw Open. 2025 Aug 13;8(8):e2526701. doi: 10.1001/jamanetworkopen.2025.26701 (PMC12351413; doi:10.1001/jamanetworkopen.2025.26701)
Supplement: Supplement 3. — Data Sharing Statement [file jamanetwopen-e2526701-s003.pdf]

Data Sharing Statement

Graham. Electric Fan Use and Hydration in Extreme Heat and Humidity. *JAMA Netw Open*. Published August 13, 2025. doi:10.1001/jamanetworkopen.2025.26701

Data
Additional Information: ACTRN12620000722998
https://anzctr.org.au/Trial/Registration/TrialReview.aspx?ACTRN=12620000722998
Data available: Yes
Data types: Deidentified participant data
How to access data: ollie.jay@sydney.edu.au
When available: With publication

Supporting Documents
Document types: None

Additional Information
Who can access the data: Researchers whose proposed use of the data has been approved
Types of analyses: For a specified purpose
Mechanisms of data availability: With a signed data access agreement
